# Emotion and Gender Typicality Cue Sexual Orientation Differently in Women and Men

**DOI:** 10.1007/s10508-020-01700-3

**Published:** 2020-05-11

**Authors:** R. Thora Bjornsdottir, Nicholas O. Rule

**Affiliations:** 1 Department of Psychology, University of Toronto, 100 St. George St., Toronto, ON M5S 3G3 Canada; 2 Present Address: Institute of Neuroscience & Psychology, University of Glasgow, 62 Hillhead St., Glasgow, G12 8QB UK

**Keywords:** Sexual orientation, Emotion, Gender, Face perception, Women

## Abstract

Heterosexual individuals tend to look and act more typical for their gender compared to gay and lesbian individuals, and people use this information to infer sexual orientation. Consistent with stereotypes associating happy expressions with femininity, previous work found that gay men displayed more happiness than straight men—a difference that perceivers used, independent of gender typicality, to judge sexual orientation. Here, we extended this to judgments of women’s sexual orientation. Like the gender-inversion stereotypes applied to men, participants perceived women’s faces manipulated to look angry as more likely to be lesbians; however, emotional expressions largely did not distinguish the faces of actual lesbian and straight women. Compared to men’s faces, women’s faces varied less in their emotional expression (appearing invariably positive) but varied more in gender typicality. These differences align with gender role expectations requiring the expression of positive emotion by women and prohibiting the expression of femininity by men. More important, greater variance within gender typicality and emotion facilitates their respective utility for distinguishing sexual orientation from facial appearance. These findings thus provide the first evidence for contrasting cues to women’s and men’s sexual orientation and suggest that gender norms may uniquely shape how men and women reveal their sexual orientation.

**Electronic supplementary material:**

The online version of this article (10.1007/s10508-020-01700-3) contains supplementary material, which is available to authorized users.

## Introduction

Impressions of another person’s sexual orientation have important downstream consequences for how people perceive and behave towards others. Despite the obvious benefits that knowing someone’s sexual orientation can afford for mate selection and group solidarity, perceptions of sexual orientation also influence life outcomes such as hiring decisions (e.g., Gross, Green, Storck, & Vanyur, 1980; Rule, Bjornsdottir, Tskhay, & Ambady, 2016). Such ramifications emphasize the value of understanding how people form impressions of sexual orientation. Most research examining cues to sexual orientation have primarily focused on gender cues, which indeed facilitate detection of sexual orientation (e.g., Freeman, Johnson, Ambady, & Rule, 2010). One set of studies expanded upon this to test how emotion expressions might cue sexual orientation (Tskhay & Rule, 2015). As that work only considered men, however, we thought it important to build upon its findings to test how emotion might cue sexual orientation in women, for whom expectations about gender and emotion differ markedly from men.

### Gender and Emotion

Gender norms outline different social roles for men and women and distinct stereotypes regarding masculinity and femininity (e.g., Eagly & Steffen, 1984; Williams & Best, 1990). These notions of masculinity and femininity come with particular expectations regarding behavior, including emotional expression. Women are expected to express positive emotion, with smiling prescribed as women’s default expression (LaFrance, Hecht, & Paluck, 2003; Stoppard & Gruchy, 1993). Men, on the other hand, are not meant to express as much emotion as women (who are stereotyped as hyperemotional), but are expected to express more dominant emotions, such as anger, to a greater extent than women (Brody, 1985; Brody & Hall, 2008; Fabes & Martin, 1991; Plant, Hyde, Keltner, & Devine, 2000). Men and women therefore have different display rules for emotion expression (which can be traced to gendered social role expectations; see Adams, Hess, & Kleck, 2015; Brody, 1997), in contrast to their lack of differences in experienced emotion (Allen & Haccoun, 1976). In line with these norms, women do smile more than men (Brody & Hall, 2008)—though not to the extent that stereotypes would suggest; such emotion expression differences reverse when women and men occupy gender-atypical social roles (e.g., men in childcare; Brody, 1997). Interestingly, men’s and women’s facial morphology actually overlaps with facial features signaling stereotypically masculine and feminine emotions, respectively (Hess, Adams, & Kleck, 2009b; Zebrowitz, Kikuchi, & Fellous, 2010). That is, the face shapes associated with happy expressions share features with those communicating female sex, whereas the face shapes associated with anger expressions overlap with those signaling male sex. These featural overlaps can further reinforce gendered expectations through bottom-up processes (additional to the top-down influence of gendered stereotypes; Adams, Nelson, Soto, Hess, & Kleck, 2012).

Gender inversion theory proposes that gay men and lesbian women have the minds of the opposite sex, thereby explaining their same-sex attraction (Katz, 2007; Lhomond, 1993). Although this explanation of homosexuality no longer enjoys popular endorsement, the reversal of gender expectations for non-heterosexuals component to gender inversion stereotypes still does. Specifically, gay men are expected to be like straight women, and lesbian women like straight men, in a plurality of their thoughts and behaviors that includes their emotional expressions (Geiger, Harwood, & Hummert, 2006; Kite & Deaux, 1987; Tskhay & Rule, 2015). Although gender inversion is an exaggerated stereotype, particularly as the association between sexual orientation and gender typicality is not always straightforward (e.g., Bailey, Bechtold, & Berenbaum, 2002), it does bear a kernel of truth: Gay and lesbian individuals indeed show more gender-nonconformity in their interests and behavior than heterosexual individuals do (e.g., Bailey & Zucker, 1995; Lippa, 2002; Pillard, 1991). This may particularly apply to lesbian women, consistent with society’s greater valuation of masculine than feminine traits (e.g., “tomboy” behavior enjoys greater tolerance than “sissy” behavior; Coyle, Fulcher, & Trübutschek, 2016; D’Augelli, Grossman, & Starks, 2008; Lippa, 2008).

### Cues to Sexual Orientation

These different stereotypes and expectations about how men and women should act unsurprisingly influence how perceivers approach decisions about others’ sexual orientation. Extant research shows that perceivers can detect others’ sexual orientation from nonverbal information with accuracy significantly exceeding chance guessing. For example, early work demonstrated that perceivers could detect sexual orientation from voice recordings (Linville, 1998) and from thin slices of nonverbal behavior in videos as short as 1 s (Ambady, Hallahan, & Conner, 1999). Later research showed that photographs of faces and even just individual facial features can allow perceivers to categorize men’s and women’s sexual orientation better than chance (Rule, Ambady, Adams, & Macrae, 2008; Rule, Ambady & Hallett, 2009a).

Consistent with gender inversion stereotypes, perceivers judge men and women with more gender atypical features or behavior as gay and lesbian from facial portraits (Dunkle & Francis, 1990), brief videos (Rieger, Linsenmeier, Gygax, Garcia, & Bailey, 2010), voice recordings (Smyth, Jacobs, & Rogers, 2003), and body movement (Johnson, Gill, Reichman, & Tassinary, 2007). Reciprocally, perceivers judge gay and lesbian individuals as more gender atypical in all of these modalities as well (e.g., Freeman et al., 2010; Lyons, Lynch, Brewer, & Bruno, 2014; Rieger et al., 2010). Thus, gendered cues facilitate detection of sexual orientation, establishing gender typicality as a valid (albeit imperfect) cue to sexual orientation cue (e.g., Freeman et al., 2010; Munson & Babel, 2007). Importantly, whereas some of these gender typicality differences may stem from biological differences (e.g., facial morphology; González-Álvarez, 2017; Skorska, Geniole, Vrysen, McCormick, & Bogaert, 2015; gait due to sexual dimorphism; Cutting, 1978), others rely on self-presentation (e.g., hairstyle; Krakauer & Rose, 2002; Rule et al., 2008; gait due to learned gender roles; see Johnson et al., 2007).

Yet gendered cues do not seem to wholly account for perceivers’ sexual orientation judgments. Using sophisticated psychophysical methods, Tskhay and Rule (2015) found that people’s mental representations of gay and straight men envisage their faces as happy and angry, respectively. Confirming this, they observed that gay and straight men displayed happy and angry facial expressions when asked to act “gay” and “straight” in the laboratory. Moreover, morphing neutral faces to look happy led participants to judge them as significantly more gay. Not only do people imagine and perceive gay men as looking happy and straight men as looking angry, actual gay and straight men seem to differ accordingly in the emotions that they show. In a final test, Tskhay and Rule demonstrated that gay and straight men looked, respectively, happier and angrier in real photographs of themselves. These emotional expression differences statistically explained others’ accurate judgments of the men’s sexual orientations. Most important, however, emotional expressions significantly predicted these sexual orientation perceptions when controlling for the men’s apparent gender typicality. Thus, emotional expression and gender typicality seem to uniquely contribute to the detection of men’s sexual orientation.

Emotional expression’s role in sexual orientation perception aligns with a wealth of research demonstrating the impact of facial emotion on perceptions of social group memberships (for review, see Bjornsdottir & Rule, 2017a). As noted above, men’s and women’s faces share features with anger and happiness expressions, respectively (Hess et al., 2009b). Importantly, this overlap impacts perception, such that an androgynous face appears more male when expressing anger and more female when expressing happiness (Adams, Nelson, Soto, Hess, & Kleck, 2012; Hess, Adams, Grammer, & Kleck, 2009a). This can be linked to emotion over generalization in face perception (Zebrowitz, 1997); however, more stereotype-driven processes can also impact perceptions of emotional faces. For example, individuals high in anti-Black prejudice more readily perceive Black faces as angry (Hugenberg & Bodenhausen, 2003). Similarly, faces expressing negative and positive emotions are judged as lower- and higher-class, respectively, which relate to (real and exaggerated) associations between social class standing and well-being (Bjornsdottir & Rule, 2017b, 2019). Facial emotion thus plays an important role in impressions of others’ social group memberships, including sexual orientation—but its part in perceptions of women’s sexual orientation remains untested.

### The Current Research

Based on the opposing associations of gay men with femininity and lesbian women with masculinity, we reasoned that parallel but complementary relations between gender typicality and emotion would explain perceptions of women’s sexual orientation (e.g., Fabes & Martin, 1991; Geiger et al., 2006; LaFrance et al., 2003). Specifically, we expected that anger would relate to perceptions of women as lesbian and that happiness would relate to perceptions of women as straight. We furthermore expected that these emotion cues would facilitate accurate perceptions of women’s sexual orientation, independent of their gender typicality, parallel to Tskhay and Rule’s (2015) findings. Thus, we tested both the utility and validity of facial emotion as a cue to women’s sexual orientation.

We began by testing emotion’s utility as a cue in Study 1. That is, do people associate anger and happiness with lesbians and straight women, respectively (regardless of whether those emotions really do characterize the two groups)? We then tested the potential validity of emotion as a cue to judgments of women’s sexual orientation in Study 2, examining whether the perception and belief that lesbian women look angry and that straight women look happy actually correspond to the expressions typical of each group. Finally, in Study 3, we replicated our results for women and Tskhay and Rule’s (2015) results for men, comparing the two to assess the relative value of emotion and gender typicality in perceptions of men’s versus women’s sexual orientation. We preregistered all three studies (Studies 1–2: https://osf.io/9k5kd, Study 3: https://osf.io/8bz3q) and make our data available on the Open Science Framework (https://osf.io/cdsqt/?view_only=7833d1572fab44f89c654e16d2358db6); we thus report how we determined our sample size, all data exclusions, all manipulations and all measures for these studies.

## Study 1

We first tested the utility of emotion cues in judgments of women’s sexual orientation. Borrowing from Tskhay and Rule’s (2015; Study 3) method, we morphed neutral face photographs to create angrier- and happier-looking versions of women’s faces, hypothesizing that participants would perceive the angry morphs as more likely to be lesbian than the original, neutral control faces and that they would perceive the happy morphs as more likely to be straight.

### Method

#### Stimuli

**Fig. 1:**
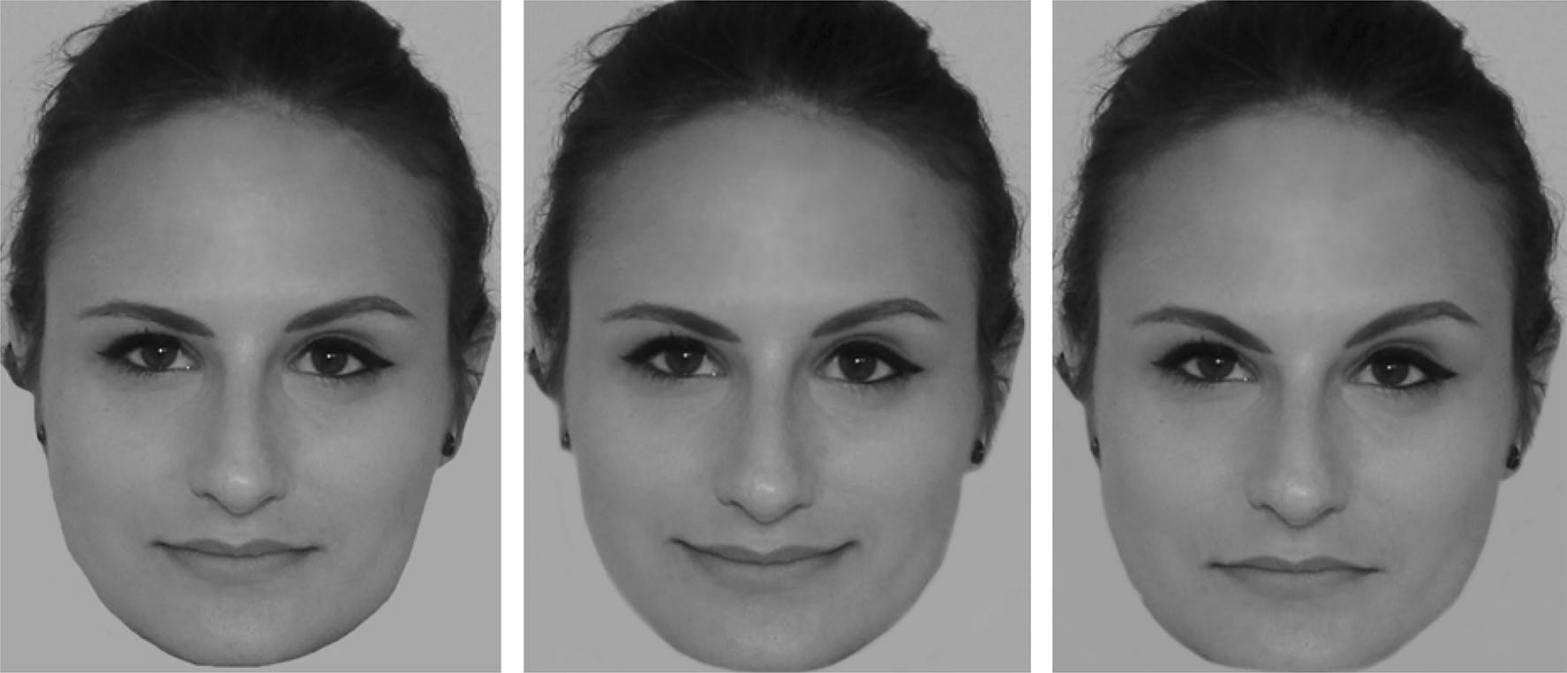
Example stimuli used in Study 1. From left: original neutral, morphed happy, and morphed angry

We collected neutrally posed facial photographs of 32 lesbians and 32 straight women (*M*_age_ = 29.61 years, SD = 11.60) from an in-house database, matched for age and ethnicity across sexual orientation as closely as possible.^1^ This number of targets afforded over 95% power to test for a main effect of target emotion in an ANOVA, anticipating the effect size reported by Tskhay and Rule (2015; Study 3A; *r*_effect size_ = .42). All targets faced the camera and none wore glasses in their photographs. We gray-scaled the images, cropped the heads from the original backgrounds (but included hair) and standardized them in height. We then used the muscle-level morph functions in FaceFilter3 (Reallusion Inc., 2013) to create angrier and happier versions of each original neutral face, following Tskhay and Rule’s (2015) procedure (see Fig. 1 for example).

#### Procedure

First, as a manipulation check, we recruited 181 participants through Amazon’s Mechanical Turk (MTurk) and randomly assigned them to rate either the original neutral photographs, happy morphs or angry morphs on either how happy or how angry they looked from 1 (*not at all*) to 7 (*very*), averaging 30 participants per rating (a sample size resulting in good inter-rater reliability in previous research, Cronbach’s *α* ≥ .80; e.g., Tskhay & Rule, 2015). This allowed us to ensure that the morphed targets’ emotions differed as intended.

Next, we recruited 90 M Turk Workers to rate the targets’ sexual orientation from 1 (*definitely lesbian*) to 8 (*definitely straight*). Each participant viewed 64 targets, with one-third displaying each emotion (neutral, happy, angry) counterbalanced across three participant groups. Participants thus rated only one version of each target, resulting in 30 participants rating each version of each target. Finally, participants provided demographic information and reported any problems viewing the images.

## Results

### Manipulation Check

We first removed the data of seven participants who reported trouble viewing the photographs, resulting in 174 participants (91 female, 82 male, 1 other; *M*_age_ = 36.40 years, SD = 12.91).^2^Both the happiness and anger ratings of all three target expressions showed excellent inter-rater reliability (Cronbach’s *α* = .92–.96); we therefore aggregated the ratings across perceivers to generate scores for each target. We then reverse-scored the mean anger ratings and averaged them with the happiness ratings to create a single (positive) emotion score, as the two ratings strongly negatively correlated across all three photographs types [*r*(62) = − .82 to − .89]. These emotion scores served as the dependent variable in a 2 (Target Sexual Orientation: Lesbian, Straight) × 3 (Target Expression: Neutral, Happy, Angry) ANOVA with repeated measures on the second factor, revealing a main effect of Target Expression, *F*(2, 124) = 159.36, *p* < .001, but not of Target Sexual Orientation, *F*(1, 62) = 0.65, *p* = .42, *r*_effect size_ = .10, 95% CI [− .15, .34], nor an interaction, *F*(2, 124) = 1.63, *p* = .20. Decomposing the target expression main effect showed that the happy morphs (*M* = 4.62, SD = 0.85) looked more positive than the original neutral faces (*M* = 4.10, SD = 0.70), *t*(63) = 14.11, *p* < .001, *r*_effect size_ = .87, 95% CI [.80, .92], which looked more positive than the angry morphs (*M* = 3.77, SD = 0.71), *t*(63) = 7.48, *p* < .001, *r*_effect size_ = .69, 95% CI [.53, .80].^3^ This confirmed that the morphing successfully altered the targets’ emotional expression.

#### Main Analysis

We removed the data of one participant who reported trouble viewing the photographs, resulting in 89 participants (41 female, 48 male; *M*_age_ = 35.80 years, SD = 12.32). The sexual orientation ratings showed excellent inter-rater reliability (Cronbach’s *α* = .91–.94), permitting us to aggregate the participants’ ratings into a mean perceived sexual orientation score for each version of each target. We then submitted these perceived sexual orientation scores to a 2 (Target Sexual Orientation: Lesbian, Straight) × 3 (Target Expression: Neutral, Happy, Angry) ANOVA, with repeated measures on the second factor, which revealed a main effect of Target Expression, *F*(2, 124) = 6.31, *p* = .002, whereby targets appeared more likely to be lesbian when displaying angry (*M* = 4.65, SD = 1.09) versus neutral (*M* = 4.82, SD = 1.10), *t*(63) = − 2.88, *p* = .005, *r*_effect size_ = − .34, 95% CI [− .55, − .10], or happy expressions (*M* = 4.86, SD = 1.04), *t*(63) = − 3.05, *p* = .003, *r*_effect size_ = − .36, 95% CI [− .56, − .12]; happy and neutral did not differ, *t*(63) = 0.60, *p* = .55, *r*_effect size_ = .08, 95% CI [− .18, .32]. A main effect of Target Sexual Orientation also emerged, *F*(1, 62) = 16.11, *p* < .001, *r*_effect size_ = .45, 95% CI [.23, .63], such that lesbian targets (*M* = 4.31, SD = 1.11) looked more likely to be lesbian than straight targets did (*M* = 5.24, SD = 0.80) regardless of their expression, *F*(2, 124) = 0.30, *p* = .74.

### Discussion

These results showed that angry-looking women seem more likely to be lesbian than neutral or happy-looking women, demonstrating the utilization of anger as a cue in inferring women’s sexual orientation. This aligns with stereotypes associating lesbians with anger (Geiger et al., 2006). But the complementary association did not emerge: Neutral and happy-looking faces appeared similarly likely to be lesbian, suggesting that perceivers may not hold the converse stereotype associating happiness with straight women.

This asymmetry fits with research showing that neutral female faces resemble happy expressions and that people expect women to smile and look happy by default (Adams et al., 2012; Hess et al., 2009b; Zebrowitz et al., 2010). Happy expressions may thus only amplify women’s happy-looking neutral state, providing no useful information about sexual orientation because women’s baseline is already near the functional ceiling. This mirrors what Tskhay and Rule (2015) found in their study with men’s faces: Happy morphs looked more gay than neutral faces and angry morphs, but the neutral and angry morphs did not significantly differ in how straight they appeared.

Different from those findings, however, we observed a main effect of sexual orientation here. The women’s actual sexual orientation remained legible across the emotion expressions. This result not only replicates previous work on the detection of sexual orientation from women’s faces but also reinforces the observation across several studies that women’s sexual orientation is more legible than men’s (e.g., Lyons et al., 2014; Rule et al., 2009b; Tabak & Zayas, 2012). Potentially more important, it may speak to the possibility that emotional expression affects perceptions of women’s sexual orientation less than men’s. Indeed, the finding could suggest that men and women diverge in the relative importance of emotion versus gender typicality in perceptions of their sexual orientation. To explore this possibility further, we next tested whether anger might also serve as a valid cue to women’s sexual orientation.

## Study 2

In Study 2, we aimed to test the validity of emotion cues in judging unmorphed neutral and naturally varying non-neutral photographs of women. Importantly, we tested whether emotion cues might contribute to sexual orientation judgments independently of gender typicality cues. We tested this using two separate stimulus sets: neutral photographs taken in the laboratory and photographs obtained from online dating profiles.

### Method

#### Stimuli

Our first stimulus set consisted of the same 64 neutral photographs used in Study 1 (importantly, there is variation in perceived emotion of even neutral faces, e.g., Adams et al., 2012). The second set represented a subset of 100 naturally varying photographs (50 lesbian, 50 straight; age range 18–35 years) collected from online dating advertisements validated in previous research (Rule et al., 2009b). We assured that the lesbian and straight women in this subset did not differ in mean attractiveness, *t*(98) = 0.03, *p* = .98, *r*_effect size_ = .003, 95% CI [− .20, 21], so that we could isolate emotion and gender typicality cues to sexual orientation without interference (given that some stereotypes portray lesbians as unattractive; Geiger et al., 2006); otherwise, we selected the photographs randomly. The targets in this set varied in their emotional expression, though most smiled. Photographs in both stimulus sets were grayscale and included the targets’ own hair and makeup. Using the two sets allowed us to test whether emotion and gender typicality cues would manifest similarly in neutral and emotional faces.

#### Procedure

We randomly assigned 240 MTurk workers to rate targets from one of the two photograph sets on one of: perceived sexual orientation (from 1 = *definitely lesbian* to 8 = *definitely straight*), happiness, anger, masculinity or femininity (all of the latter four from 1 = *not at all* to 7 = *very*) in a fully between-subjects design. To conserve resources, we used the happiness and anger ratings of the neutral targets collected in Study 1, recruiting roughly 30 participants to complete each of the remaining eight conditions. As before, this participant sample allowed for good inter-rater reliability. After rating the targets, all participants reported their demographic information and any problems viewing the images.

### Results

We removed the data of 12 participants who reported trouble viewing the stimuli, leaving 228 perceivers with complete data (110 female, 118 male; *M*_age_ = 36.03 years, SD = 11.50). High inter-rater reliabilities permitted us to average the perceivers’ ratings to create scores for each target on every dimension (Cronbach’s *α* = .82–.98). As in Study 1, the happiness and anger ratings strongly negatively correlated in both photograph sets [*r*(62) = − .85, *r*(98) = − .93], so we combined the reverse-scored mean anger ratings with the mean happiness ratings to form a single (positive) emotion score for each target. The masculinity and femininity mean ratings also strongly correlated [*r*(62) = − .95; *r*(98) = − .94], so we likewise averaged femininity and reverse-scored masculinity into a single gender typicality score for each target.

**Fig. 2:**
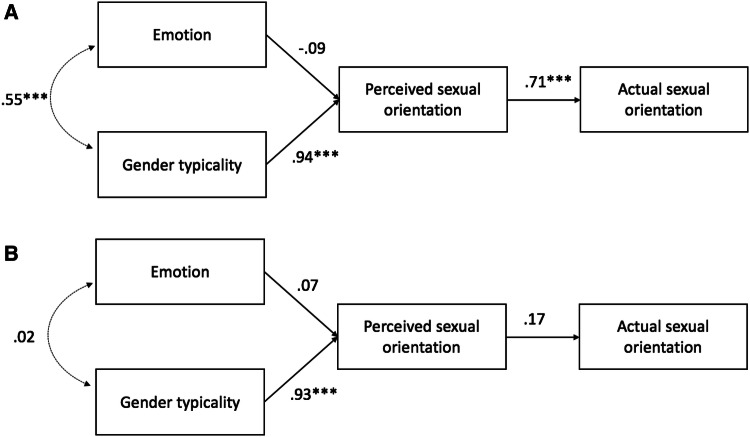
Unconstrained path models measuring the influence of (positive) emotion and gender typicality on women’s perceived and actual sexual orientation from **a** neutrally posed and **b** naturally varying targets in Study 2. ***p < .001

We entered these scores into a target-level path model (see Fig. 2) in which emotion and gender typicality predicted perceived sexual orientation, which subsequently predicted actual sexual orientation (coded 0 = lesbian, 1 = straight). Following Tskhay and Rule (2015), we estimated the model using a weighted least squares estimator due to the binary nature of the outcome variable (actual sexual orientation). The model showed good fit for the naturally varying dating profile photographs, SRMR = .04, RMSEA = .04, 90% CI [0, .21], TLI = .999, but poor fit for the neutrally posed in-lab photographs, SRMR = .10, RMSEA = .25, 90% CI [.11, .41], TLI = .86. Replicating previous work (Freeman et al., 2010), gender typicality strongly predicted perceived sexual orientation for both the neutrally posed, *β* = .94, *z* = 11.24, *p* < .001, and naturally varying targets, *β* = .93, *z* = 28.56, *p* < .001, such that less gender-typical (i.e., more masculine) women looked more likely to be lesbian. Furthermore, perceived sexual orientation predicted actual sexual orientation for the neutrally posed targets, *β* = .71, *z* = 4.18, *p* < .001, though not significantly for the naturally varying targets, *β* = .17, *z* = 1.21, *p* = .23.^4^

**Fig. 3:**
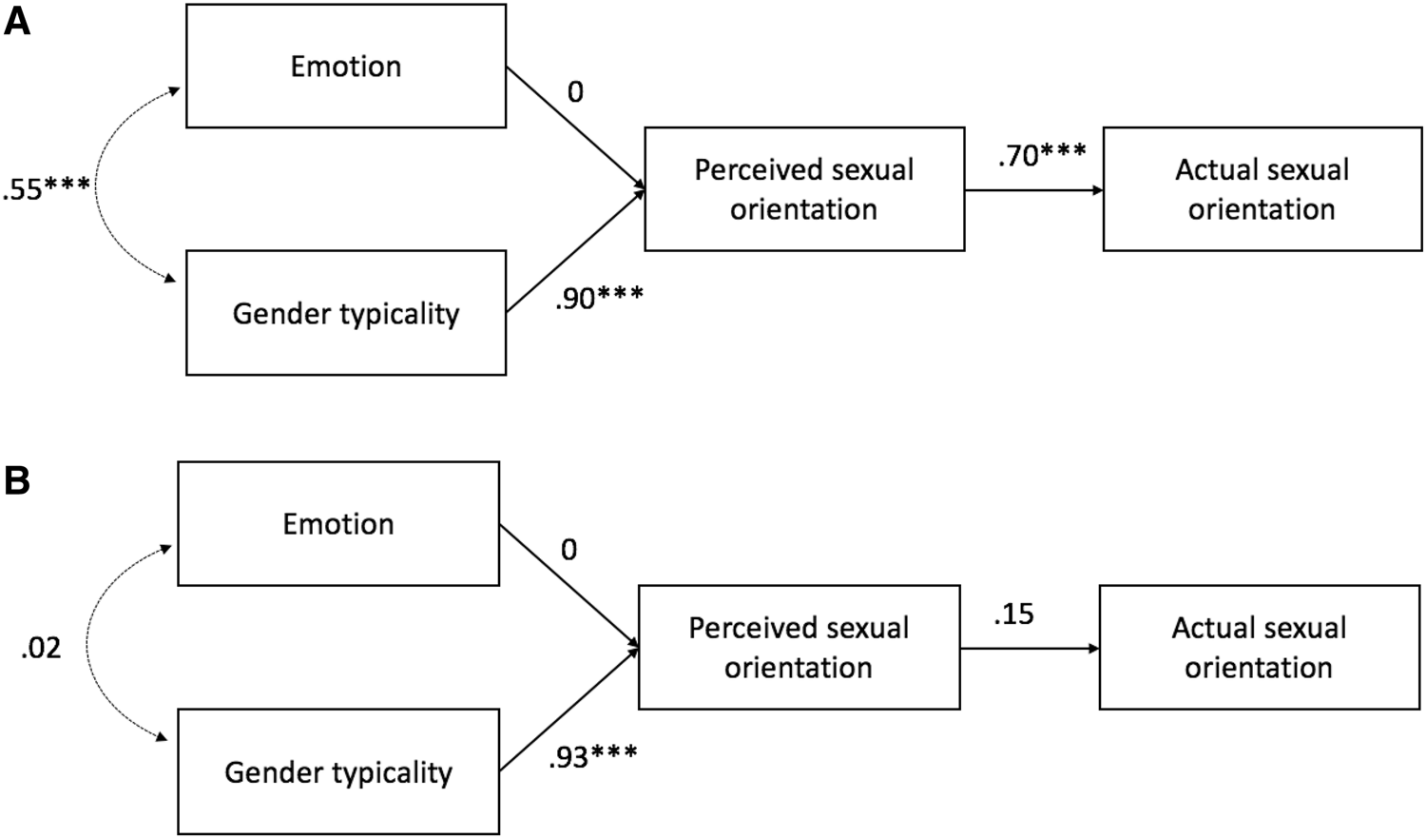
Constrained path models for women’s **a** neutrally posed and **b** naturally varying targets in Study 2. ***p < .001

Contrary to our hypothesis, however, emotion did not significantly predict perceived sexual orientation for either the neutrally posed, *β* = − .09, *z* = − 1.27, *p* = .20, or the naturally varying targets, *β* = .07, *z* = 1.80, *p* = .07. To better understand what enabled detection of the targets’ sexual orientations, we therefore constrained the emotion path to 0 (Fig. 3), again observing good fit for the naturally varying targets, SRMR = .04, RMSEA = .09, 90% CI [0, .21], TLI = .995 and poor fit for the neutrally posed targets, SRMR = .09, RMSEA = .21, 90% CI [.09, .35], TLI = .90. Finally, we compared the fit of the constrained and the unconstrained models to test whether emotion’s contribution to the model was negligible. Likelihood ratio tests showed no significant differences between the constrained and original models for either the neutrally posed, *χ*^2^(1) = 1.56, *p* = .21 or naturally varying targets, *χ*^2^(1) = 3.25, *p* = .07, favoring the constrained models for their parsimony. Emotion therefore did not promote accurate perception of the targets’ sexual orientation.

## Discussion

Here, we found that gender typicality, but not emotion, contributed to the detection of women’s sexual orientation. This contrasts with Tskhay and Rule’s (2015) findings that both gender typicality and emotion cues independently contributed to impressions of sexual orientation from men’s faces. These results moreover appear to contradict the findings of Study 1, in which perceivers judged angrier-looking women as more likely to be lesbian than neutral and happier-looking women; however, we digitally manipulated the targets’ emotions there but relied on natural variations in expression here.

Given the structural similarities between women’s neutral expressions and happiness and society’s proscription against women expressing negative emotions, these null results for emotion might stem from insufficient negative emotion among both the neutrally posed and naturally varying targets (Brody & Hall, 2008; Hess et al., 2009b; LaFrance et al., 2003). Preliminary comparisons of the variance in ratings of the women’s faces here and in the men’s faces in Tskhay and Rule’s (2015, Study 4A) data indeed showed less variation in the women’s emotion scores but more variation in their gender typicality scores compared to the men. Emotion may therefore constitute a more functional cue for judging men’s sexual orientation, whereas gender typicality may better serve judgments of women’s faces. To test this idea directly, we replicated the present tests in Study 3 using both male and female targets to allow us to directly compare the relative value of emotion and gender typicality in perceptions of sexual orientation between the two groups.

## Study 3

Women’s emotional expression did not relate to perceptions of their sexual orientation in Study 2, contrasting with what Tskhay and Rule (2015) found for men. To better understand this difference, we repeated Study 2 using both male and female targets to compare the two here. We hypothesized that emotion would facilitate detection only of men’s sexual orientation but that gender typicality would facilitate detection of both men’s and women’s sexual orientation. We furthermore predicted that men’s emotional expressions would vary more than women’s, but that women’s gender typicality would vary more than men’s.

### Method

#### Stimuli

As in Study 2, we used neutrally posed photographs taken in the laboratory and naturally varying photographs collected from online dating profiles. We gathered the neutrally posed photographs of every lesbian woman and gay man for which we had usable photographs (i.e., those with clear photographs not wearing glasses) in our in-house database and matched them with an equal number of straight counterparts by age and ethnicity as closely as possible (*n*s = 66 lesbians, 132 gay men, 66 straight women, 132 straight men; *M*_age_ = 24.25 years, SD = 8.18). We gray-scaled the photographs, standardized them in height and cropped them to the top of the head (including hair), bottom of the chin and extremes of the ears.

We furthermore obtained photographs of 94 lesbians, 98 straight women, 95 gay men and 86 straight men (aged 18–35 years) originally collected from online dating profiles and validated in previous research that cropped the faces from their original backgrounds, gray-scaled them and standardized them in size (see Rule, 2011; Rule et al., 2008, 2009b).

#### Procedure

The procedure followed that of Study 2: An average of 30 MTurk workers were randomly assigned to rate either the women’s or men’s neutrally posed or naturally varying dating profile photographs on one of perceived sexual orientation, happiness, anger, masculinity or femininity (total *N* = 761). Participants rated the faces using the same scales described above, except that we modified the anchors for perceptions of the men’s sexual orientations to 1 (*definitely gay*) and 8 (*definitely straight*). Due to the large number of targets, perceivers rating the men’s neutral photographs rated a random subset of half of the targets to avoid fatigue. As in Studies 1 and 2, participants ended the study by providing demographic information and reporting any problems viewing the target photographs.

### Results

We excluded the data of 32 participants who reported problems viewing the stimuli, resulting in 729 participants (405 female, 324 male; *M*_age_ = 38.69 years, SD = 12.39). High inter-rater reliabilities permitted us to average the perceivers’ ratings into scores for each target on every trait (Cronbach’s *α* = .80–.98). As in Study 2, we reverse-scored the anger ratings to combine them with happiness into (positive) emotion and combined femininity and masculinity (with femininity reverse-scored for male targets and masculinity reverse-scored for female targets) into gender typicality scores [range *r*(262) = − .80 to (130) = − .94, across all stimulus sets]. We then tested separate path models for each of the four stimulus sets.

**Fig. 4:**
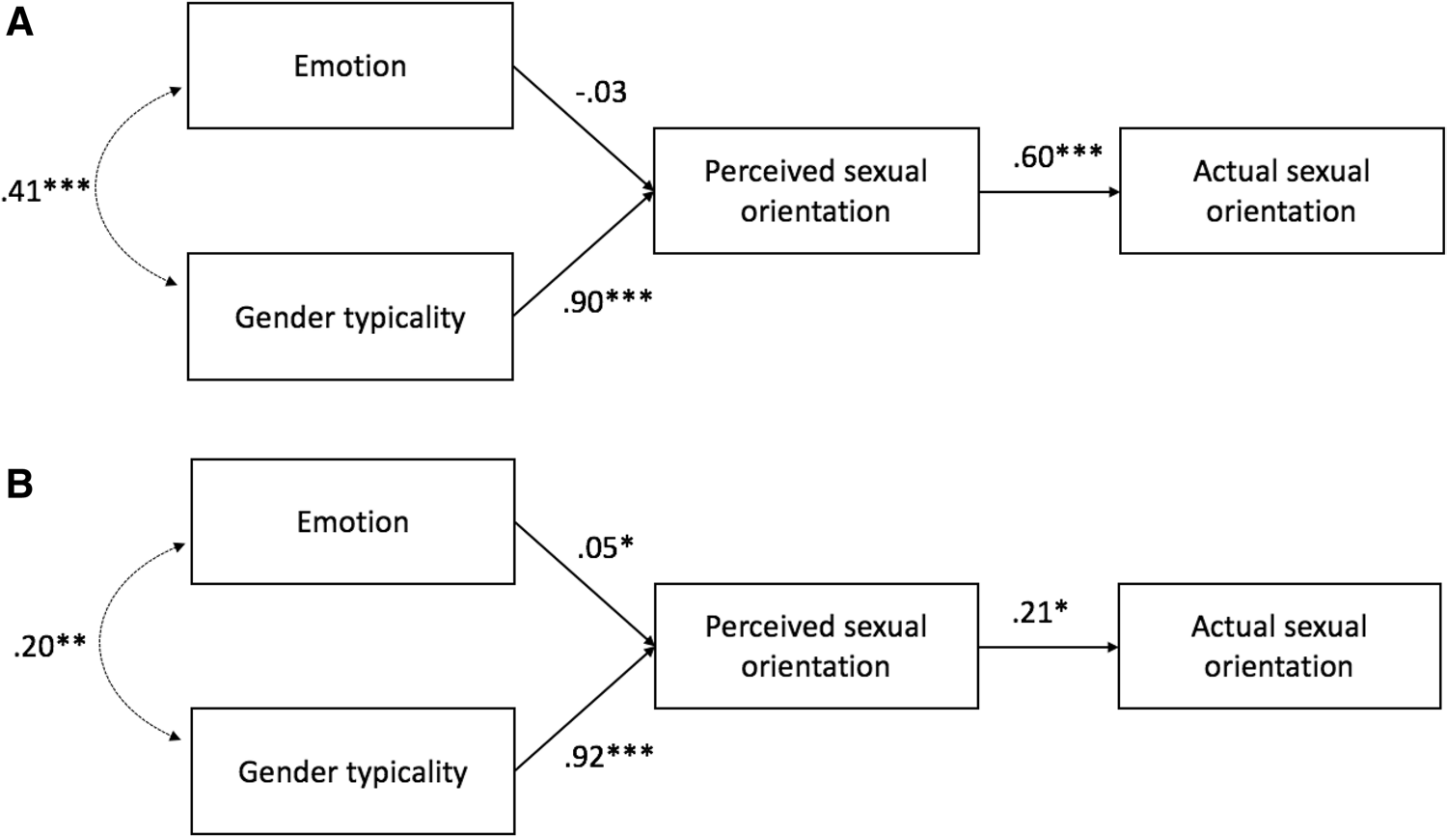
Unconstrained path models measuring the influence of (positive) emotion and gender typicality on women’s perceived and actual sexual orientation from **a** neutrally posed and **b** naturally varying targets in Study 3. *p < .05; **p < .01; ***p < .001

**Fig. 5:**
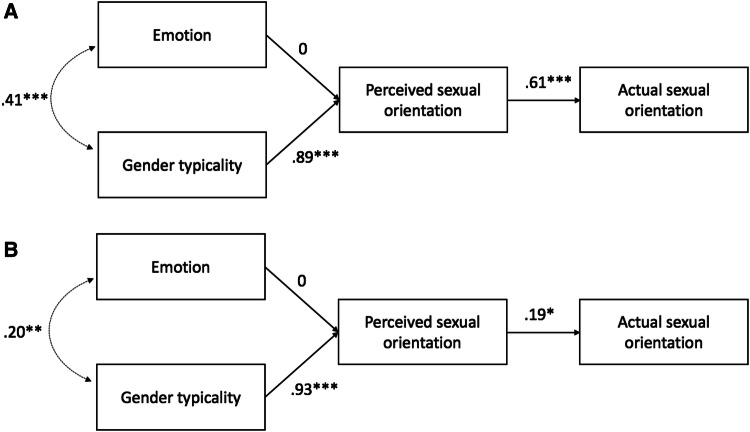
Constrained path models for women’s **a** neutrally posed and **b** naturally varying targets in Study 3. *p < .05; **p < .01; ***p < .001

**Fig. 6:**
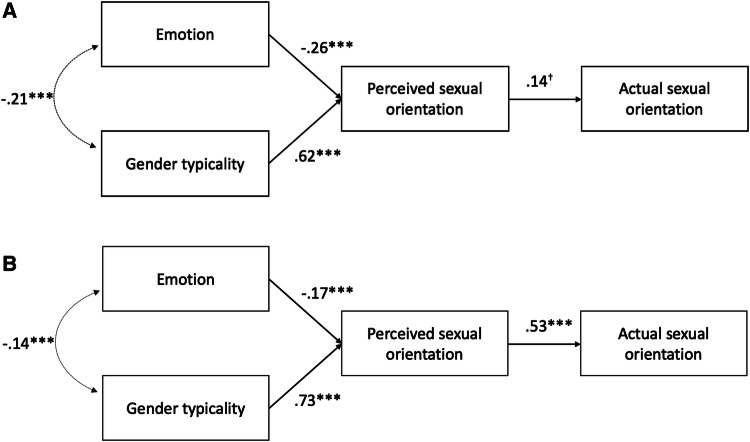
Path models measuring the influence of (positive) emotion and gender typicality on men’s perceived and actual sexual orientation from **a** neutrally posed and **b** naturally varying targets in Study 3. ^†^*p* = .05; ***p < .001

We again tested a model in which emotion and gender typicality independently predicted perceived sexual orientation, which in turn predicted actual sexual orientation (see Figs. 4, 5, 6).^5^ This model fit the data well for the women’s neutrally posed photographs, SRMR = .05, RMSEA = .000, 90% CI [0, .16], TLI = 1, women’s naturally varying photographs, SRMR = .02, RMSEA = .08, 90% CI [0, .18], TLI = .995, men’s neutrally posed photographs, SRMR = .01, RMSEA = .09, 90% CI [.01, .18], TLI = .97 and men’s naturally varying photographs, SRMR = .001, RMSEA = .000, 90% CI [0, .08], TLI = 1.^6^

Replicating Study 2, for women’s neutrally posed photographs, gender typicality significantly predicted perceived sexual orientation, *β* = .90, *z* = 21.43, *p* < .001, but emotion did not, *β* = − .03, *z* = − 0.78, *p* = .44. Perceived sexual orientation moreover significantly predicted targets’ actual sexual orientation, *β* = .60, *z* = 5.59, *p* < .001. More masculine-looking women therefore appeared more likely to be lesbian, which facilitated accurate impressions of their sexual orientation. Among the naturally varying photographs, gender typicality again significantly predicted women’s perceived sexual orientation, *β* = .92, *z* = 34.13, *p* < .001, but, in contrast with the neutral photographs, so did emotion (a marginally significant result in Study 2), *β* = .05, *z* = 2.13, *p* = .03. Perceived sexual orientation furthermore predicted actual sexual orientation, *β* = .21, *z* = 2.14, *p* = .03, supporting our suspicion that tightly constraining attractiveness undermined this association in Study 2 (Fig. 4).^7^ Thus, for the naturally varying photographs, more masculine and angrier women looked more likely to be lesbian, which enabled detection of their actual sexual orientation.

Finally, constraining the path between emotion and perceived sexual orientation did not significantly change model fit for the neutrally posed photographs, SRMR = .05, RMSEA = .000, 90% CI [0, .13], TLI = 1.00), *χ*^2^(1) = 0.61, *p* = .43, favoring the simpler constrained model because it predicted targets’ sexual orientation more parsimoniously (Fig. 5). The constrained model fits the data well for the naturally varying photographs, SRMR = .03, RMSEA = .10, 90% CI [.03, .18], TLI = .99, but significantly less well than the full model did, *χ*^2^(1) = 4.54, *p* = .03. Emotion thus contributed meaningfully (and independently of gender typicality) to impressions of sexual orientation from women’s naturally varying photographs but contributed negligibly to impressions from women’s neutral photographs.

For men, we replicated Tskhay and Rule’s (2015) findings with both the neutrally posed and naturally varying targets. Gender typicality predicted perceived sexual orientation for both the neutrally posed, *β* = .62, *z* = 16.64, *p* < .001, and naturally varying photographs, *β* = .73, *z* = 16.97, *p* < .001 (though not as strongly as for the women). Moreover, in contrast to the neutrally posed women, emotion independently predicted perceived sexual orientation for both neutrally posed, *β* = − .26, *z* = − 5.26, *p* < .001, and naturally varying men’s photographs, *β* = − .17, *z* = − 3.68, *p* < .001, such that happier men appeared gay. Perceived sexual orientation in turn predicted actual sexual orientation (coded 0 = gay, 1 = straight) among the neutrally posed, *β* = .14, *z* = 1.93, *p* = .05, and naturally varying photographs, *β* = .53, *z* = 6.83, *p* < .001 (Fig. 6). Thus, men who looked happier and less gender typical (i.e., more feminine) seemed more likely to be gay, which facilitated perceivers’ accurate impressions of their sexual orientation.

Next, we compared the variance in emotion and gender typicality ratings between the men’s and women’s faces using Levene’s test for equality of variances. Among the naturally varying targets, men’s emotion scores (*M* = 5.10, SD = 1.19) varied more than women’s (*M* = 5.41, SD = 0.81), *F*(1, 371) = 33.72, *p* < .001, *r*_effect size_ = .29, 95% CI [.19, .38], but women’s gender typicality scores (*M* = 4.91, SD = 0.94) varied more than men’s (*M* = 5.02, SD = 0.62), *F*(1, 371) = 20.98, *p* < .001, *r*_effect size_ = .23, 95% CI [.13, .33]. Among the neutrally posed targets, men’s (*M* = 3.94, SD = 0.69) and women’s emotion scores (*M* = 4.02, SD = 0.77) varied similarly, *F*(1, 394) = 2.93, *p* = .09, *r*_effect size_ = .09, 95% CI [− .01, .19], but women’s gender typicality scores (*M* = 4.67, SD = 0.98) again varied more than men’s (*M* = 5.19, SD = .71), *F*(1, 394) = 7.28, *p* = .007, *r*_effect size_ = .13, 95% CI [.04, .23].

Finally, as an exploratory test, we meta-analytically compared the effect sizes for the covariance between emotion and gender typicality (Rosenthal & Rubin, 1982). This analysis revealed that emotion and gender typicality covaried more strongly for women’s neutrally posed photographs, compared to the naturally varying women, *z* = 2.04, *p* = .04, neutrally posed men, *z* = 2.07, *p* = .04, and naturally varying men, *z* = 2.55, *p* = .01. No other significant differences emerged, all *z*s ≤ 0.74, *p*s ≥ .46.

### Discussion

These results confirmed our hypothesis that the cues to sexual orientation differ between women and men. As in Study 2, gender typicality predicted accurate perceptions of women’s sexual orientation. Emotion did not predict perceived sexual orientation for women’s neutral photographs, paralleling the results of Study 2, but did significantly contribute to a small degree to detecting the sexual orientation from women’s naturally varying photographs, diverging from Study 2. In contrast, both emotion and gender typicality predicted the detection of men’s sexual orientation across both photograph types, replicating Tskhay and Rule’s (2015) earlier findings. Meta-analytic comparisons of the associations between emotion and gender typicality with perceived sexual orientation showed that gender typicality (when controlling for emotion) predicted perceptions of women’s sexual orientation significantly more strongly than men’s (neutral photographs: *z* = 5.58, *p* < .001; naturally varying photographs: *z* = 6.56, *p* < .001), whereas emotion (when controlling for gender typicality) predicted perceptions of men’s sexual orientation more strongly than women’s (neutral photographs: *z* = 2.72, *p* = .007; naturally varying photographs: *z* = 1.40, *p* = .16). Thus, people seem to principally rely on differences in men’s emotional expression and differences in women’s gender typicality to reliably infer their sexual orientations.

Indeed, differences in the variability of emotion and gender typicality within the men’s and women’s faces helped to explain these differences. Gender typicality varied significantly more in women’s faces than in men’s faces, supporting its utility as a diagnostic cue. In other words, the greater variation in women’s gender typicality makes it easier to identify differences between individual targets along this dimension, which then seem to systematically cluster according to their sexual orientation (i.e., straight women are more gender typical and lesbian women are less gender typical). Emotion complementarily varied more in men’s faces than in women’s faces, though understandably not when they posed neutral expressions. Among the neutral photographs, however, emotion and gender typicality covaried more strongly among women than men, helping explain why emotion did not independently predict perceptions of sexual orientation from women’s neutral photographs, but did from men’s.

These differences align with how men and women normatively express emotion and gender. People expect women to express positive emotion as a default. Because women may suffer social repercussions for failing to do so (e.g., Stoppard & Gunn Gruchy, 1993), they might reasonably limit their range of emotional expression to only positive displays, particularly in an evaluative context such as a dating profile (for similar findings in yearbooks, see Dodd, Russell, & Jenkins, 1999). Men, on the other hand, face similar constraints from traditional notions about masculinity that might provoke them to restrict their gender atypicality more than women do (Sánchez, Greenberg, Liu, & Vilain, 2009). Indeed, women report more childhood gender nonconformity than men do (regardless of sexual orientation); moreover, gender nonconformity relates to self-reported anxiety and distress only for men (Lippa, 2008; Skidmore, Linsenmeier, & Bailey, 2006) and men are penalized for feminine behavior (e.g., Berdahl, 2007; Rudman & Mescher, 2013). Women thus seem to have more leeway with their gender expression and may also have more socially acceptable options for manipulating the gender typicality of their appearance compared to men (e.g., through hairstyle and cosmetics; Krakauer & Rose, 2002; Rule et al., 2008). Altogether, our findings suggest that social norms differentially affect how men and women signal their sexual orientation to others.

## General Discussion

These studies provide the first evidence that the facial cues to men’s and women’s sexual orientation differ. Gender typicality facilitates accurate impressions of sexual orientation from both men’s and women’s faces, such that less gender-typical individuals look more likely to be gay and lesbian. Emotional expression also informs the detection of sexual orientation from men’s faces, however: Men who express more happiness seem more likely to be gay. For women, emotional expression provides a small contribution to detecting sexual orientation—but only for naturally varying photographs. Indeed, the results of Study 1 suggest that perceivers will use emotion cues to infer women’s sexual orientation if those cues are present.

Specifically, perceivers will employ stereotypes to judge angry-looking women as more likely to be lesbian. Neutral and happy-looking women’s faces look similarly likely to be straight, however, aligning with previous work demonstrating that female facial morphology shares features with expressions of happiness and that society expects women to look positive as their default expression (i.e., to smile; Adams et al., 2012; Briton & Hall, 1995; Hess et al., 2009b; LaFrance et al., 2003). Indeed, the results of Studies 2 and 3 indicate that women primarily express positive emotion, and often with insufficient variance for anger to serve as a cue to sexual orientation. It therefore appears that social norms surrounding women’s emotional expressions may render emotion a fairly unhelpful cue to women’s sexual orientation, particularly when women’s faces are neutral.

Although society generally expects men to express less emotion than women, it does allow them to express more anger (e.g., Brody, 1985; Fabes & Martin, 1991). And whereas women face negative social consequences when violating gendered expectations of emotional expression (i.e., failing to express positive emotion), men report smiling just as often as women when experiencing happiness and receive positive evaluations when smiling (Deutsch, LeBaron, & Fryer, 1987; Hess et al., 2000; Stoppard & Gunn Gruchy, 1993). Thus, although society stereotypes women as hyperemotional, its norms require them to appear positive; and although society stereotypes men as stoic, it permits them to express both anger and happiness, affording them a broader range of emotional expression (though not unconstrained lability).

Moreover, although gender typicality cued sexual orientation for both men and women, it contributed more strongly to impressions of women’s sexual orientation. Women accordingly showed more variable gender typicality than men did, perhaps related to the greater flexibility that society allows them for gender expression (i.e., gender nonconforming boys are evaluated more negatively than gender nonconforming girls, and gender nonconformity relates to anxiety and distress in men but not in women; Kwan et al., 2019; Lippa, 2008; Martin, 1990; Skidmore et al., 2006). Curiously, women’s permission to violate gender norms does not translate into doing so by transgressing emotional display rules—or at least not by much. This may result from the greater overall value that society places on masculinity, which sympathizes with women’s emulation of men to an extent, but prohibits women’s displays of high-power negative emotions such as anger and aggression (e.g., Feinman, 1981; Fischer, Rodriguez Mosquera, Van Vianen, & Manstead, 2004). Indeed, this aligns with work showing that children’s gender atypicality is evaluated differently by gender: Boys are penalized more for appearing feminine than are girls for appearing masculine, whereas girls are evaluated more negatively for certain masculine behaviors (i.e., loud and rough play) compared to boys with certain feminine behaviors (i.e., quiet and gentle play; Blakemore, 2003). Together, these differences suggest that social norms surrounding men’s and women’s emotion and gender expression may shape how they manifest and express their sexual orientation.

These patterns held across different types of photographs. Tskhay and Rule (2015) used only photographs from dating profiles in their study demonstrating that both gender typicality and emotion cue men’s sexual orientation. We replicated this result both with standardized neutral photographs and with dating profile photographs that varied in their emotional expression. We furthermore observed primarily consistent results across the two types of photographs for women. Our findings thus held across different stimulus types and largely replicated as we increased the number of targets, supporting their external validity and generalizability.

Future research could expand on our work to examine how gender typicality and emotion cue men’s and women’s sexual orientation across cultures, where gendered norms of emotional expression vary (Fischer & Manstead, 2000). Such efforts might also consider target ethnicity, given featural overlaps with emotional expressions (Zebrowitz et al., 2010) and as sexual dimorphism also varies across ethnic groups (Hopder, Finklea, Winkielman, & Huber, 2014; Wells, 2012). Although our neutral targets here varied in ethnicity (suggesting that the pattern of results may generalize across several different ethnic groups), most were Caucasian, necessitating future systematic testing of the generalizability of how ethnicity, emotion, gender and sexual orientation might interact. Extant work indicates that sexual orientation is detected equivalently across both target and perceiver ethnicity (at least when judging men’s sexual orientation; Rule, 2011; Rule, Ishii, Ambady, Rosen, & Hallett, 2011) and suggests that perceivers use gender typicality as a cue regardless of target ethnicity (though perhaps rely more heavily on it for certain groups; Johnson & Ghavami, 2011). Future work could expound these findings to specifically test how gender typicality and emotion expression relate to perceived and actual sexual orientation among targets of varying intersections of gender and ethnicity.

Future work can also more directly test the role of biological differences in appearance (e.g., facial structure; Skorska et al., 2015) versus self-presentation (e.g., hairstyle, makeup, emotion expression; Krakauer & Rose, 2002; Rule et al., 2008) in influencing gender typicality and perceived emotion, and thereby informing perceptions of sexual orientation. For example, research could compare gender typicality, emotion and sexual orientation judgments of the same individuals photographed both with and without gendered self-presentation cues (e.g., makeup removed, hair masked), and both with neutral and spontaneously posed facial expressions.

Overall, these data provide a greater understanding of how people perceive and form impressions about sexual orientation and point to the importance of investigating the intersection of social groups and dimensions. Here, we found that social norms may affect how men and women express their sexual orientation, and the cues that perceivers use to reliably detect it. Thus, social norms may affect distinct sexual minority groups differently, stimulating questions about how the expectations particular to each group might affect subsequent social consequences.

## Electronic supplementary material

Below is the link to the electronic supplementary material.

Supplementary material 1 (DOCX 274 kb)

## Footnotes

1 Targets either reported a strong preference for women over men (or vice versa) on a continuous measure of sexual orientation or dichotomously categorized themselves as lesbian or straight. Across all studies, targets from our in-house database were 51.7% Caucasian, 25.7% East or Southeast Asian, 4.8% African, 4.3% mixed-race, 3.9% South Asian, 3.5% Hispanic, 1.7% Middle Eastern, 0.7% First Nations, 0.7% Pacific Islander, and 3.0% unreported ethnicity; targets collected from online dating profiles were all Caucasian.

2 Across all studies, perceivers were 69.5% Caucasian, 9.3% African, 6.0% mixed-race, 5.1% Hispanic, 3.8% East Asian, 1.9% South Asian, 1.7% Southeast Asian, 0.6% Middle Eastern, 0.4% Native American, 0.2% Pacific Islander, and 1.5% unreported ethnicity.

3 We found the same pattern of results when modeling the happiness and anger ratings separately: The angry morphs looked angrier than the neutral faces, and the happy morphs looked happier than the neutral faces.

4 This positive but nonsignificant association could have resulted from preselecting targets based on attractiveness, which strongly relates to gender typicality (i.e., femininity) in women’s faces and validly relates to sexual orientation (Freeman et al., 2010; Perrett et al., 1998). This preselection may also explain the lack of covariance between emotion and gender typicality in this sample.

5 Please see the Supplementary Material for correlation matrices, as well as exploratory mediation models with emotion and gender typicality as mediators between actual and perceived sexual orientation.

6 We removed the data for three male targets included erroneously (one ostensibly straight and two ostensibly gay) because their reported sexual orientations did not match the inclusion criteria (i.e., they were either bisexual or did not report their sexual orientation).

7 Although we could not deliberately match the attractiveness of the straight and lesbian women’s faces here as we had in Study 2 because we used all of the available faces rather than a subset, 21 MTurk Workers’ ratings of their attractiveness nevertheless showed that the lesbian and straight targets’ mean attractiveness scores (Cronbach’s *α* = .94) did not significantly differ: *t*(190) = 1.37, *p* = .17, *r*_effect size_ = .10, 95% CI [− .04, .24].
