# Supplementary material for: Emotion and Gender Typicality Cue Sexual Orientation Differently in Women and Men
Source: Arch Sex Behav. 2020 May 11;49(7):2547–60. doi: 10.1007/s10508-020-01700-3 (PMC7497461; doi:10.1007/s10508-020-01700-3)
Supplement: Supplementary file 1 — Supplementary material 1 (DOCX 274 kb) [file 10508_2020_1700_MOESM1_ESM.docx]

Supplementary Material

**Study 3 Mediation Results and Discussion**

As an exploratory analysis, we also tested Emotion and Gender Typicality as mediators of the relation between actual and perceived sexual orientation (see also Table S1 for correlation matrices). We conducted multiple mediation analyses using bootstrapping (with 5000 bootstrap resamples to provide stable estimates) to estimate the size and standard errors of both indirect pathways (Preacher & Hayes, 2008) and coded actual sexual orientation as 0 = gay/lesbian, 1 = straight.

For neutral women’s photos, Gender Typicality functioned as a significant mediator, *a_1_b_1_* = .54, *SE* = .14, 95% CI [.28, .82], but Emotion did not, *a_2_b_2_* = -.001, *SE* = .01, 95% CI [-.02, .02] (see Figure S1). Altogether, the total indirect effect for the set of mediators was significant, *f* = .54, SE = .14, 95% CI [.28, .82]. Furthermore, although both the total effect, *c* = .83, *SE* = .15, 95% CI [.53, 1.13], and the direct effect, *c’* = .29, *SE* = .08, 95% CI [.14, .45], were significant, the magnitude of the direct effect decreased substantially compared to the total effect.

Similarly, for the naturally varying women’s photos, Gender Typicality significantly mediated the relationship between actual and perceived sexual orientation, *a_1_b_1_* = .29, *SE* = .14, 95% CI [.02, .56], but Emotion did not, *a_2_b_2_* = .02, *SE* = .01, 95% CI [-.01, .05] (see Figure S1). The total indirect effect was furthermore significant, *f* = .31, SE = .14, 95% CI [.04, .58], and though the total effect was significant, *c* = .38, *SE* = .15, 95% CI [.10, .68], the direct effect was not, *c’* = .07, *SE* = .06, 95% CI [-.04, .18], indicating full mediation.

In contrast, for men’s neutral photos, Gender Typicality did not serve as a mediator, *a_1_b_1_* = .02, *SE* = .05, 95% CI [-.06, .12], whereas Emotion did, *a_2_b_2_* = .05, *SE* = .02, 95% CI [.01, .09] (see Figure S2). Altogether, the total indirect effect for the two mediators was not significant, *f* = .07, SE = .05, 95% CI [-.03, .17], suggesting that both mediators were not necessary in explaining the relationship between actual and perceived sexual orientation. Moreover, although the total effect was significant, *c* = .15, *SE* = .07, 95% CI [.004, .30], the direct effect was not, *c’* = .08, *SE* = .05, 95% CI [-.02, .18], indicating full mediation.

Finally, similar to the women’s photos, for men’s naturally varying photos, whereas Gender Typicality was a significant mediator, *a_1_b_1_* = .25, *SE* = .06, 95% CI [.14, .38], Emotion was not, *a_2_b_2_* = .02, *SE* = .02, 95% CI [-.01, .05] (see Figure S2). The total indirect effect was furthermore significant, *f* = .27, SE = .06, 95% CI [.15, .40], as were both the total effect, *c* = .49, *SE* = .08, 95% CI [.34, .64], and the direct effect, *c’* = .22, *SE* = .06, 95% CI [.11, .33]—though the magnitude of the direct effect was substantially lower than that of the total effect.

Altogether, these results suggest that gender typicality primarily explains the relationship between women’s perceived and actual sexual orientation, regardless of photo type, echoing the pattern found in our primary path analyses. Furthermore, although cue use was the same across the two samples (with only gender typicality independently predicting perceived sexual orientation), cue validity varied somewhat: Gender typicality related to actual sexual orientation for both stimulus sets, but emotion related to actual sexual orientation only for the naturally varying targets (indeed, where there was greater variance in emotion expression).

For men, emotion mediated the relationship between perceived and actual sexual orientation for neutrally posed photos, whereas gender typicality served as the mediator for naturally varying photos. Cue use was consistent across these two stimulus sets, such that both emotion and gender typicality independently predicted perceived sexual orientation. In contrast, cue validity varied between the stimulus sets, with actual sexual orientation significantly relating only to emotion for the neutrally posed photos and only to gender typicality for the naturally varying photos. Together, this suggests that although perceivers may employ the same cues to judge sexual orientation, regardless of the type of photo (e.g., neutral, varying in emotion expression), how these cues relate to actual sexual orientation may vary according to stimulus type. Whether this pattern replicates across other stimulus sets and why men’s sexual orientation may relate to emotion in neutral faces and gender typicality in emotionally varying faces remain questions worthy of future investigation.

Table S1

*Intercorrelations for Study 3 Targets’ Actual and Perceived Sexual Orientation, Emotion, and Gender Typicality*

|  | Actual sexual orientation | Perceived sexual orientation | Emotion |
| --- | --- | --- | --- |
| Women’s neutral photos |  |  |  |
| Actual sexual orientation | — |  |  |
| Perceived sexual orientation | .43*** | — |  |
| Emotion | .06 | .35*** | — |
| Gender Typicality | .33*** | .89*** | .41*** |
| Women’s naturally varying photos |  |  |  |
| Actual sexual orientation | — |  |  |
| Perceived sexual orientation | .18* | — |  |
| Emotion | .18* | .24** | — |
| Gender Typicality | .15* | .92*** | .20** |
| Men’s neutral photos |  |  |  |
| Actual sexual orientation | — |  |  |
| Perceived sexual orientation | .12* | — |  |
| Emotion | -.17** | -.38*** | — |
| Gender Typicality | .03 | .67*** | -.21*** |
| Men’s naturally varying photos |  |  |  |
| Actual sexual orientation | — |  |  |
| Perceived sexual orientation | .42*** | — |  |
| Emotion | -.08 | -.28*** | — |
| Gender Typicality | .33*** | .75*** | -.14^†^ |

*Note.* ^†^ *p < .10; ** *p* < .05; **** *p* < .01; **** p* < .001.

A

*
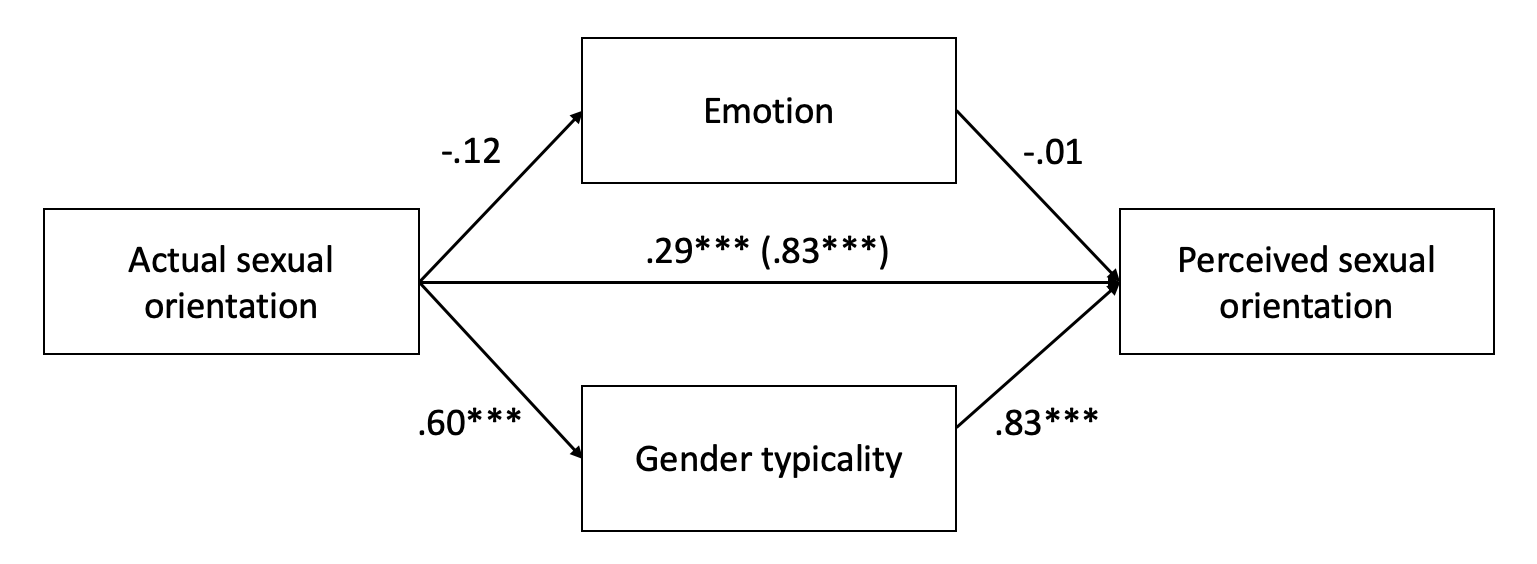
*

B

*
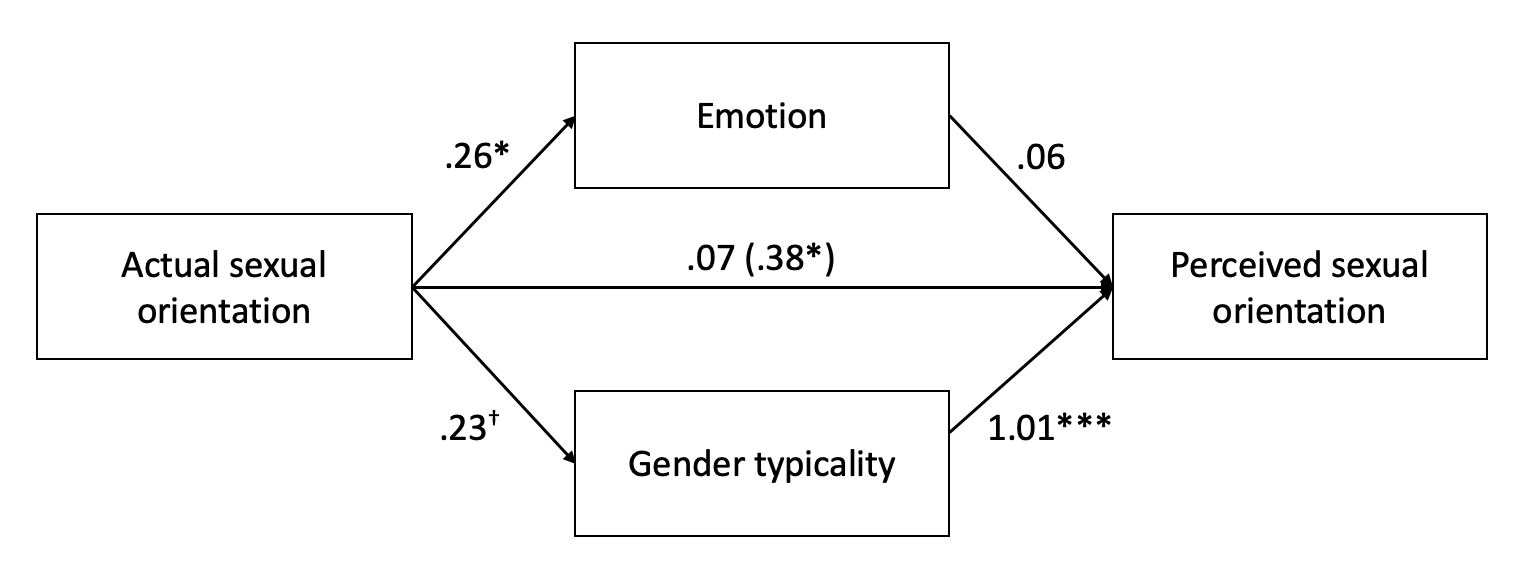
*

*Figure S1.* Mediation models with (Positive) Emotion and Gender Typicality as mediators of the relation between actual and perceived sexual orientation from women’s (A) neutrally posed and (B) naturally varying photos in Study 3. ^†^ *p < .10; ** *p* < .05; **** p* < .001.

A

***
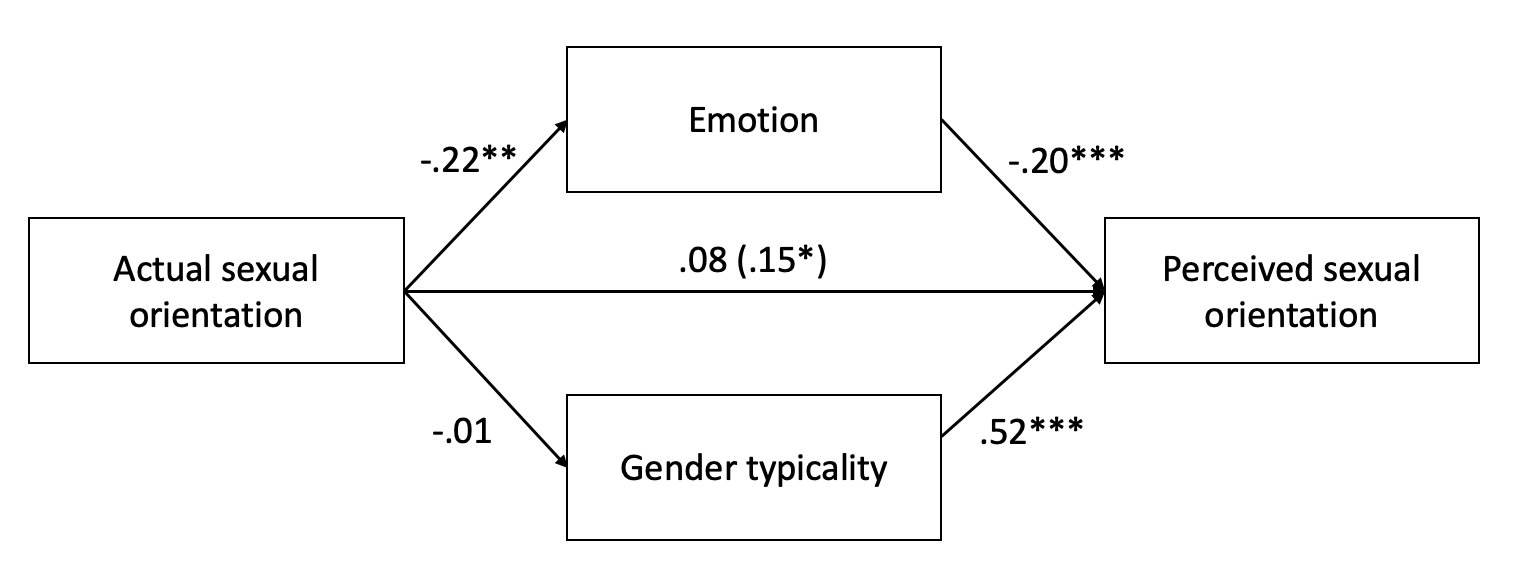
***

B

***
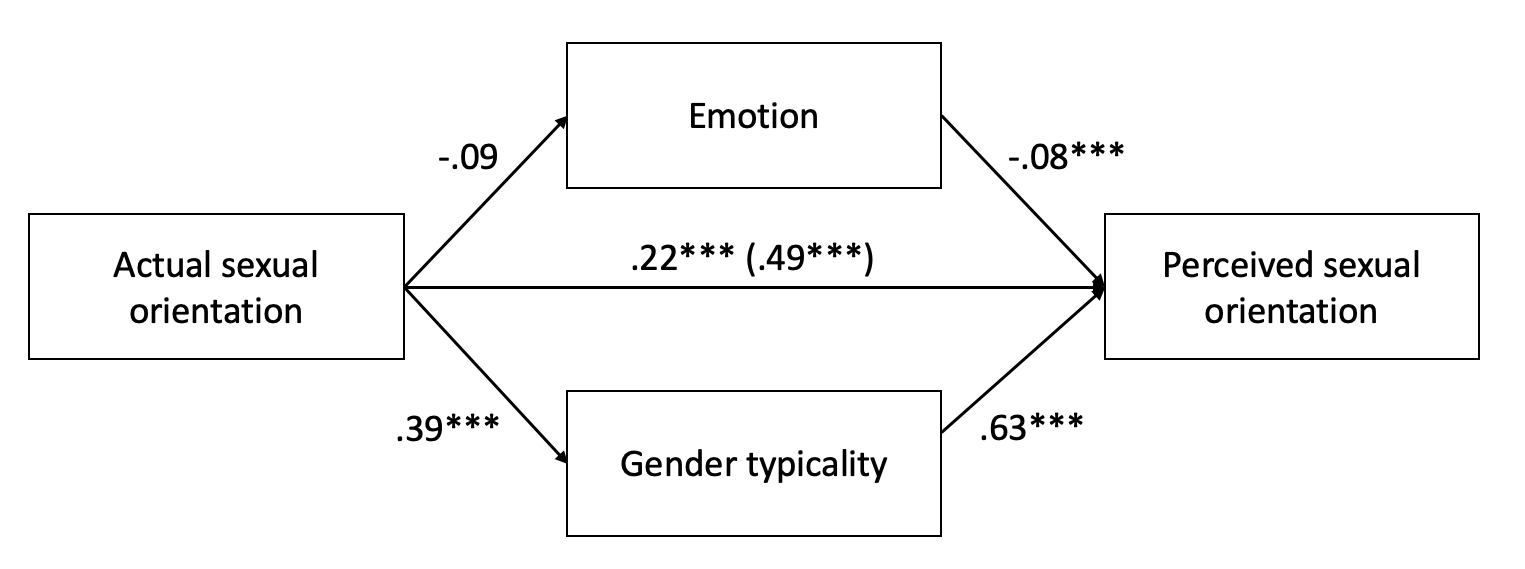
***

*Figure S2.* Mediation models with (Positive) Emotion and Gender Typicality as mediators of the relation between actual and perceived sexual orientation from men’s (A) neutrally posed and (B) naturally varying photos in Study 3. **** *p* < .01; **** p* < .001.
